# Supplementary material for: Brain-derived neurotrophic factor genetic polymorphism rs6265 and creativity
Source: PLoS One. 2023 Sep 13;18(9):e0291397. doi: 10.1371/journal.pone.0291397 (PMC10499242; doi:10.1371/journal.pone.0291397)
Supplement: Attachment — Submitted filename: Review PONE-D-23-07754.pdf [file pone.0291397.s001.pdf]

The Manuscript “Brain-derived neurotrophic factor genetic polymorphism and creativity” reports potential implications of the *BDNF* Val66met polymorphism on parameters of creativity – taken by the Compound Remote Associate (CRA) task – in health young adults.

BDNF is a neurotrophin whose gene is common to all mammals and possible all invertebrates that have nervous system; whereas the Val66met polymorphism only occurs in humans. Descriptive data have shown a higher prevalence of the BDNF polymorphism in subpopulations in psychopathological disorders, such as depression, compared to general populations; as opposed to a lack of a direct effect of the BDNF val66met polymorphism on neurodegenerative-related processes.

With that been said, in my opinion this study bring up an interesting perspective for the research on the BDNF val66met polymorphism.

English language requires no revision (from a non-native speaker perspective).

The Methods and Statistics were accurate and are well described and applied.

I suggest that the authors create a Figure – Study’s design describing the methods in a Timeline/schematics to help the readers visualizing the procedures, evaluation tools (and controls) adopted.

Results appropriately presented. I know that the number of homozygote individuals for the Met-containing allele is very small and that they appear mostly always combined to the heterozygotes in the studies. However, I have to ask whether have the authors tested running the stats with the groups spared by homozygous vs heterozygous. There has been recently demonstrated an effect of the Val66met polymorphism on BDNF gene expression function in human (reference below), and results led me to put it on perspective.

I believe the Discussion is clear and unbiased, and provides a didactic synthesis of what was pinpointed in the intersection of two major fields - behavioral Neuroscience and Molecular neurobiology. I miss a some updated references though, when it comes to the intra-cellular mechanisms demonstrably affected by the BDNF val66met polymorphism up to date (reference below).

de Assis GG, Hoffman JR, Bojakowski J, Murawska-Ciałowicz E, Cięszczyk P, Gasanov E V. (2021) The Val66 and Met66 Alleles-Specific Expression of BDNF in Human Muscle and Their Metabolic Responsivity. Frontiers in Molecular Neuroscience, 14, 1–12.

de Assis GG, Hoffman JR (2022) The BDNF Val66Met Polymorphism is a Relevant, But not Determinant, Risk Factor in the Etiology of Neuropsychiatric Disorders – Current Advances in Human Studies: A Systematic Review. Brain Plasticity, 8, 133–142.
